# Crystal structure of di­aqua­bis­(4-*tert*-butyl­benzoato-κ*O*)bis­(nicotinamide-κ*N* ^1^)cobalt(II) dihydrate

**DOI:** 10.1107/S2056989016008689

**Published:** 2016-06-03

**Authors:** Gülçin Şefiye Aşkın, Hacali Necefoğlu, Safiye Özkaya, Raziye Çatak Çelik, Tuncer Hökelek

**Affiliations:** a Department of Physics, Hacettepe University, 06800 Beytepe, Ankara, Turkey; b Department of Chemistry, Kafkas University, 36100 Kars, Turkey; c International Scientific Research Centre, Baku State University, 1148 Baku, Azerbaijan; d Scientific and Technological Application and Research Center, Aksaray University, 68100, Aksaray, Turkey

**Keywords:** crystal structure, cobalt(II), transition metal complexes, benzoic acid, nicotinamide

## Abstract

The asymmetric unit of the mononuclear cobalt complex, [Co(C_11_H_13_O_2_)_2_(C_6_H_6_N_2_O)_2_(H_2_O)_2_]·2H_2_O, contains one half of the complex mol­ecule, one coordinating and one non-coordinating water mol­ecule, one 4-*tert*-butyl­benzoate (TBB) ligand and one nicotinamide (NA) ligand; the Co atom lies on an inversion centre. All ligands coordinating to the Co atom are monodentate. The four nearest O atoms around the Co atom form a slightly distorted square-planar arrangement, with the distorted octa­hedral coordination completed by the two pyridine N atoms of the NA ligands at distances of 2.1638 (11) Å. The coordinating water mol­ecules are hydrogen bonded to the carboxyl O atoms [O ⋯ O = 2.6230 (17) Å], enclosing an *S*(6) hydrogen-bonding motif, while inter­molecular O—H⋯O hydrogen bonds link two of the non-coordinating water mol­ecules to the coordinating water mol­ecules and NA anions. The dihedral angle between the planar carboxyl­ate group and the adjacent benzene ring is 29.09 (10)°, while the benzene and pyridine rings are oriented at a dihedral angle of 88.53 (4)°. In the crystal, O—H⋯O and N—H⋯O hydrogen bonds link the mol­ecules, enclosing *R*_2_^2^(8), *R*_2_^2^(10) and *R*_4_^4^(12) ring motifs, forming layers parallel to (001). The C and H atoms of the *tert*-butyl group of the TBB ligand are disordered over two sets of sites with an occupancy ratio of 0.631 (5):0.369 (5).

## Abstract 2

The asymmetric unit of the monomeric cobalt complex, [Co(C_11_H_13_O_2_)_2_(C_6_H_6_N_2_O)_2_(H_2_O)_2_]·2H_2_O, contains one half of the complex mol­ecule, one coordinating and one non-coordinating water, one 4-*tert*-butyl­benzoate (TBB) ligand and one nicotinamide (NA) ligand; the Co atom lies on an inversion centre. The coordinating water mol­ecules are hydrogen bonded to the carboxyl O atoms [O ⋯ O = 2.6230 (17) Å], enclosing an *S*(6) hydrogen-bonding motif, while inter­molecular O—H⋯O hydrogen bonds link two of the non-coordinating water mol­ecules to the coordinating water mol­ecules and NA anions. In the crystal, O—H⋯O and N—H⋯O hydrogen bonds link the mol­ecules, enclosing 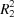
(8), 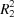
(10) and 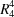
(12) ring motifs, forming layers parallel to (001).

## Chemical context   

Nicotinamide (NA) is one form of niacin. A deficiency of this vitamin leads to loss of copper from the body: a condition known as pellagra disease. Victims of pellagra show unusually high serum and urinary copper levels (Krishnamachari, 1974 ▸). The NA ring is the reactive part of nicotinamide adenine dinucleotide (NAD) and its phosphate (NADP), which are the major electron carriers in many biological oxidation-reduction reactions (You *et al.*, 1978 ▸). The nicotinic acid derivative *N*,*N*-di­ethyl­nicotinamide (DENA) is an important respiratory stimulant (Bigoli *et al.*, 1972 ▸). The structures of some complexes obtained from the reactions of transition metal(II) ions with NA as ligand, *e.g*. [Ni(NA)_2_(C_7_H_4_ClO_2_)_2_(H_2_O)_2_] [(II); Hökelek *et al.*, 2009 ▸], [Zn(NA)_2_(C_7_H_4_NO_4_)_2_]_*n*_ [(III); Aşkın *et al.*, 2015*a* ▸] and [Co(NA)_2_(C_8_H_4_NO_2_)_2_(H_2_O)_2_] [(IV); Aşkın *et al.*, 2015*b* ▸], have been determined previously. In all complexes, the NA and benzoate ligands coordinate the transition metal(II) ions as monodentate ligands.

Transition metal complexes with biochemical mol­ecules show inter­esting physical and/or chemical properties, through which they may find applications in biological systems (Antolini *et al.*, 1982 ▸). Some benzoic acid derivatives, such as 4-amino­benzoic acid, have been extensively reported in coordination chemistry, as bifunctional organic ligands, due to the varieties of their coordination modes (Chen & Chen, 2002 ▸; Amiraslanov *et al.*, 1979 ▸; Hauptmann *et al.*, 2000 ▸).

The structure–function–coordination relationships of the aryl­carboxyl­ate ion in Co^II^ complexes of benzoic acid deriv­atives may change depending on the nature and position of the substituent groups on the benzene ring, the nature of the additional ligand mol­ecule or solvent, and the pH and temperature of synthesis (Shnulin *et al.*, 1981 ▸; Nadzhafov *et al.*, 1981 ▸; Antsyshkina *et al.*, 1980 ▸; Adiwidjaja *et al.*, 1978 ▸). When pyridine and its derivatives are used instead of water mol­ecules, the structure is completely different (Catterick *et al.*, 1974 ▸). In this context, we synthesized a Co^II^-containing compound with 4-*tert*-butylbenzoate (TBB) and NA ligands, namely di­aqua­bis­(4-*tert*-butyl­benzoato-κ*O*)bis­(nicotinamide-κ*N* ^1^)cobalt(II) dihydrate, [Co(C_11_H_13_O_2_)_2_(C_6_H_6_N_2_O)_2_ 
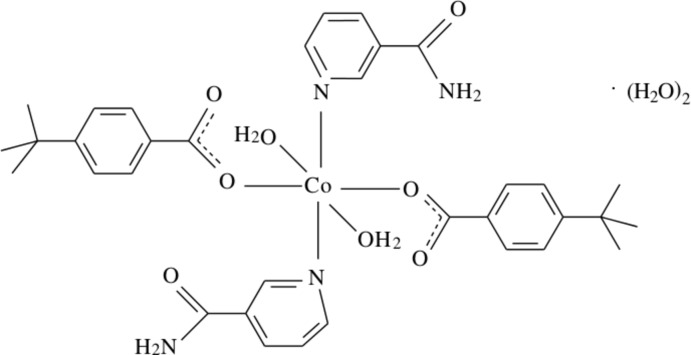


## Structural commentary   

**Figure 1:**
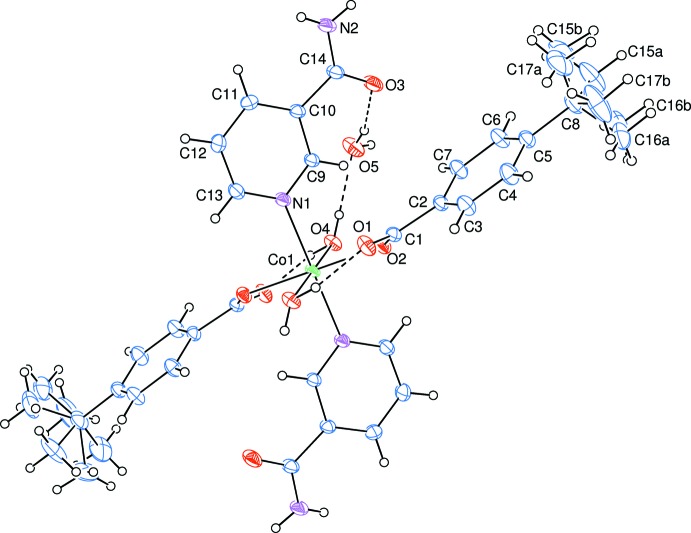
The mol­ecular structure of the title complex with the atom-numbering scheme. Displacement ellipsoids are drawn at the 40% probability level. Intra- and inter­molecular O—H⋯O hydrogen bonds are shown as dashed lines.

The asymmetric unit of the crystal structure of the mononuclear title complex contains one 4-*tert*-butyl­benzoate (TBB) and one nicotinamide (NA) ligand together with one coordinating and one non-coordinating water mol­ecule, all ligands coordinating in a monodentate manner (Fig. 1 ▸).

In the title complex, the two carboxyl­ate O atoms (O2 and O2^i^) of the two symmetry-related monodentate TBB anions and the two symmetry-related coordinating water O atoms (O4 and O4^i^) around the Co1 (site symmetry 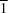) atom form a slightly distorted square-planar arrangement, while the slightly distorted octa­hedral coordination sphere is completed by the two pyridine N atoms (N1 and N1^i^) of the two symmetry-related monodentate NA ligands in the axial positions [symmetry code: (i) −*x*, −*y*, −*z*] (Fig. 1 ▸).

The near equalities of the C1—O1 [1.2526 (17) Å] and C1—O2 [1.2702 (16) Å] bonds in the carboxyl­ate groups indicate delocalized bonding arrangements, rather than localized single and double bonds. The Co—O bond lengths are 2.1104 (11) Å (for water oxygens) and 2.1252 (9) Å (for benzoate oxygens) and the Co—N bond length is 2.1638 (11) Å, close to standard values. The Co1—O2—C1—C2 torsion angle [−163.00 (9)°] causes a slight downward tilt of the ligand.

The dihedral angle between the planar carboxyl­ate group (O1/O2/C1) and the adjacent benzene (C2–C7) ring is 29.09 (10)°, while the benzene and pyridine (N1/C9–C13) rings are oriented at a dihedral angle of 88.53 (4)°.

Intra­molecular O—H_w_⋯O_c_ (w = water, c = carboxyl­ate) hydrogen bonds (Table 1 ▸) link the coordinating water mol­ecules to the TBB anions, enclosing *S*(6) hydrogen-bonding motifs, while inter­molecular O—H_w_⋯O_w_ and O—H_w_⋯O_na_ (na = nicotinamide) hydrogen bonds link two of the non-coordinating water mol­ecules to the coordinating water mol­ecules and NA anions (Fig. 1 ▸).

## Supra­molecular features   

**Figure 2:**
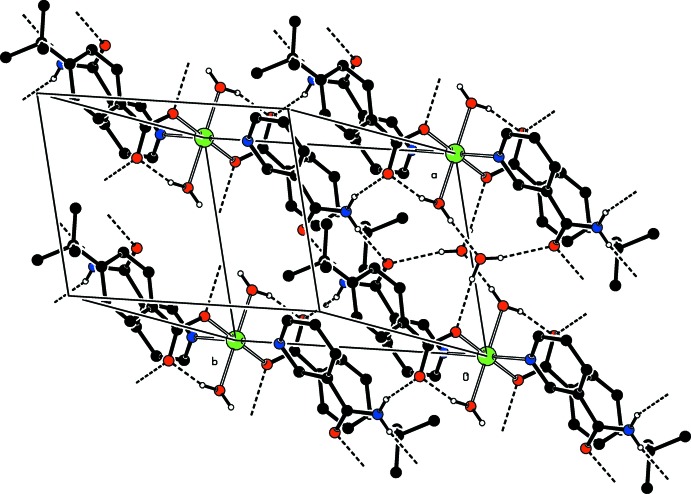
A partial view of the crystal packing of the title compound. Inter­molecular O—H_w_⋯O_w_, O—H_w_⋯O_NA_, O—H_w_⋯O_c_, N—H_NA_⋯O_c_ and N—H_NA_⋯O_NA_ (w = water, c = carboxyl­ate and NA = nicotinamide) hydrogen bonds, enclosing 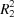
(8), 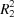
(10) and 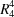
(12) ring motifs, are shown as dashed lines (see Table 1 ▸). For clarity, only the major disorder component and H atoms involved in hydrogen bonding are shown.

**Figure 3:**
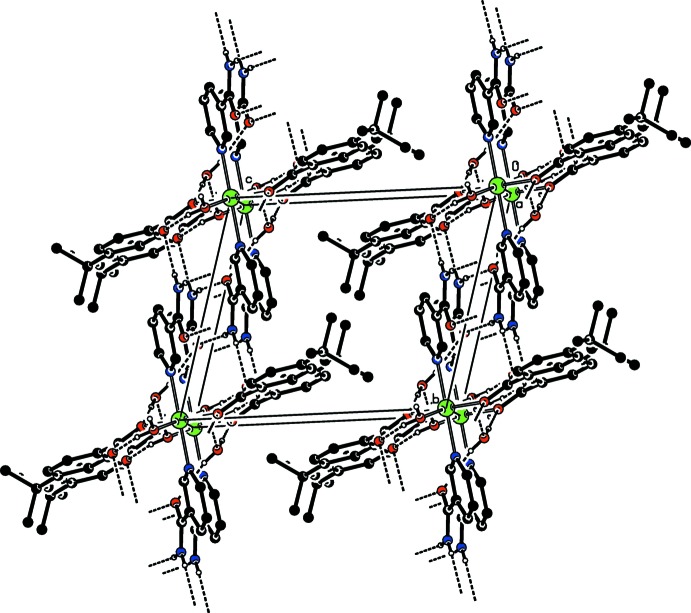
Part of the crystal structure viewed down [100]. Intra- and inter­molecular O—H⋯O and N—H⋯O hydrogen bonds are shown as dashed lines. For clarity, only the major disorder component and H atoms involved in hydrogen bonding are shown.

In the crystal, O—H_w_⋯O_c_, N—H_na_⋯O_c_ and N—H_na_⋯O_na_ hydrogen bonds (Table 1 ▸) link the mol­ecules, enclosing 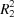
(8), 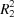
(10) and 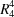
(12) ring motifs (Fig. 2 ▸), forming layers parallel to (001) (Fig. 3 ▸).

## Synthesis and crystallization   

The title compound was prepared by the reaction of CoSO_4_·7H_2_O (1.41 g, 5 mmol) in water (75 ml) and nicotinamide (1.22 g, 10 mmol) in water (25 ml) with sodium 4-*tert*-butyl­benzoate (2.00 g, 10 mmol) in water (250 ml). The mixture was filtered and set aside to crystallize at ambient temperature for five days, giving pink single crystals.

## Refinement   

Experimental details including the crystal data, data collection and refinement are summarized in Table 2 ▸. Atoms H21 and H22 (for NH_2_), H41, H42, H51 and H52 (for H_2_O) were located in a difference Fourier map and were refined freely. The C-bound H atoms were positioned geometrically, with C—H = 0.93 and 0.96 Å for aromatic and methyl H atoms, respectively, and constrained to ride on their parent atoms, with *U*_iso_(H) = *k* × *U*_eq_(C), where *k* = 1.5 for methyl H atoms and *k* = 1.2 for aromatic H atoms. During the refinement process the disordered *t*-butyl group atoms were refined with major:minor occupancy ratios of 0.631 (5):0.369 (5).

## Supplementary Material

Crystal structure: contains datablock(s) I, global. DOI: 10.1107/S2056989016008689/pk2579sup1.cif

Structure factors: contains datablock(s) I. DOI: 10.1107/S2056989016008689/pk2579Isup2.hkl

CCDC reference: 1482507

Additional supporting information: crystallographic information; 3D view; checkCIF report

## Figures and Tables

**Table 1:** Hydrogen-bond geometry (Å, °)

*D*—H⋯*A*	*D*—H	H⋯*A*	*D*⋯*A*	*D*—H⋯*A*
N2—H21⋯O1^i^	0.82 (2)	2.15 (2)	2.935 (2)	159 (2)
N2—H22⋯O3^ii^	0.85 (2)	2.07 (2)	2.907 (2)	166 (2)
O4—H41⋯O1^iii^	0.87 (3)	1.79 (3)	2.6230 (17)	160 (3)
O4—H42⋯O5	0.84 (2)	2.01 (2)	2.852 (2)	176.4 (19)
O5—H51⋯O3	0.82 (3)	2.14 (3)	2.942 (2)	164 (3)
O5—H52⋯O2^iv^	0.87 (3)	2.17 (3)	3.0331 (19)	175 (3)

Symmetry codes: (i) 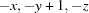; (ii) 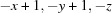; (iii) 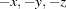; (iv) 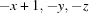.

**Table 2:** Experimental details

Crystal data	
Chemical formula	[Co(C_11_H_13_O_2_)_2_(C_6_H_6_N_2_O)_2_(H_2_O)_2_]·2H_2_O
*M*_r_	729.69
Crystal system, space group	Triclinic, *P* 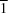
Temperature (K)	296
*a*, *b*, *c* (Å)	7.9608 (5), 10.0679 (6), 12.3007 (7)
α, β, γ (°)	72.087 (2), 74.841 (3), 78.660 (3)
*V* (Å^3^)	898.17 (9)
*Z*	1
Radiation type	Mo *K*α
μ (mm^−1^)	0.54
Crystal size (mm)	0.45 × 0.34 × 0.28

Data collection	
Diffractometer	Bruker *SMART* BREEZE CCD
Absorption correction	Multi-scan (*SADABS*; Bruker, 2012 ▸)
*T*_min_, *T*_max_	0.80, 0.86
No. of measured, independent and observed [*I* > 2σ(*I*)] reflections	19515, 4491, 4226
*R*_int_	0.024
(sin θ/λ)_max_ (Å^−1^)	0.669

Refinement	
*R*[*F* ^2^ > 2σ(*F* ^2^)], *wR*(*F* ^2^), *S*	0.036, 0.101, 1.04
No. of reflections	4491
No. of parameters	276
No. of restraints	156
H-atom treatment	H atoms treated by a mixture of independent and constrained refinement
Δρ_max_, Δρ_min_ (e Å^−3^)	0.57, −0.20

Computer programs: *APEX2* and *SAINT* (Bruker, 2012 ▸), *SHELXS97* (Sheldrick, 2008 ▸), *SHELXL2014* (Sheldrick, 2015 ▸), *ORTEP-3 for Windows* and *WinGX* (Farrugia, 2012 ▸) and *PLATON* (Spek, 2009 ▸).

## supplementary crystallographic information

### Crystal data

**Table:**

[Co(C_11_H_13_O_2_)_2_(C_6_H_6_N_2_O)_2_(H_2_O)_2_]·2H_2_O	*Z* = 1
*M**_r_* = 729.69	*F*(000) = 385
Triclinic, *P*1	*D*_x_ = 1.349 Mg m^−^^3^
*a* = 7.9608 (5) Å	Mo *K*α radiation, λ = 0.71073 Å
*b* = 10.0679 (6) Å	Cell parameters from 9878 reflections
*c* = 12.3007 (7) Å	θ = 2.4–28.4°
α = 72.087 (2)°	µ = 0.54 mm^−^^1^
β = 74.841 (3)°	*T* = 296 K
γ = 78.660 (3)°	Prism, pink
*V* = 898.17 (9) Å^3^	0.45 × 0.34 × 0.28 mm

### Data collection

**Table:**

Bruker SMART BREEZE CCD diffractometer	4226 reflections with *I* > 2σ(*I*)
φ and ω scans	*R*_int_ = 0.024
Absorption correction: multi-scan (*SADABS*; Bruker, 2012)	θ_max_ = 28.4°, θ_min_ = 1.8°
*T*_min_ = 0.80, *T*_max_ = 0.86	*h* = −10→10
19515 measured reflections	*k* = −13→13
4491 independent reflections	*l* = −16→16

### Refinement

**Table:**

Refinement on *F*^2^	156 restraints
Least-squares matrix: full	Hydrogen site location: mixed
*R*[*F*^2^ > 2σ(*F*^2^)] = 0.036	H atoms treated by a mixture of independent and constrained refinement
*wR*(*F*^2^) = 0.101	*w* = 1/[σ^2^(*F*_o_^2^) + (0.0645*P*)^2^ + 0.2513*P*] where *P* = (*F*_o_^2^ + 2*F*_c_^2^)/3
*S* = 1.04	(Δ/σ)_max_ < 0.001
4491 reflections	Δρ_max_ = 0.57 e Å^−^^3^
276 parameters	Δρ_min_ = −0.20 e Å^−^^3^

### Special details

**Table:**

Geometry. All esds (except the esd in the dihedral angle between two l.s. planes) are estimated using the full covariance matrix. The cell esds are taken into account individually in the estimation of esds in distances, angles and torsion angles; correlations between esds in cell parameters are only used when they are defined by crystal symmetry. An approximate (isotropic) treatment of cell esds is used for estimating esds involving l.s. planes.
Refinement. Refined as a 2-component inversion twin.

### Fractional atomic coordinates and isotropic or equivalent isotropic displacement parameters (Å^2^)

**Table:**

	*x*	*y*	*z*	*U*_iso_*/*U*_eq_	Occ. (<1)
Co1	0.0000	0.0000	0.0000	0.02676 (9)	
O1	−0.15897 (14)	0.14320 (12)	0.21665 (11)	0.0417 (3)	
O2	0.08538 (13)	0.01768 (10)	0.14429 (9)	0.0325 (2)	
O3	0.43372 (17)	0.35986 (12)	−0.03048 (13)	0.0529 (3)	
O4	0.26754 (14)	−0.04949 (12)	−0.07344 (10)	0.0366 (2)	
H41	0.256 (4)	−0.078 (3)	−0.131 (2)	0.074 (8)*	
H42	0.350 (3)	−0.001 (2)	−0.0935 (19)	0.054 (6)*	
O5	0.54025 (18)	0.12406 (16)	−0.13794 (15)	0.0559 (3)	
H51	0.523 (4)	0.181 (3)	−0.100 (2)	0.071 (8)*	
H52	0.645 (4)	0.079 (3)	−0.140 (2)	0.072 (7)*	
N1	0.01589 (15)	0.22272 (12)	−0.07666 (10)	0.0299 (2)	
N2	0.3410 (2)	0.58855 (14)	−0.10004 (14)	0.0436 (3)	
H21	0.269 (3)	0.651 (2)	−0.1300 (18)	0.045 (5)*	
H22	0.419 (3)	0.607 (2)	−0.0731 (18)	0.050 (5)*	
C1	−0.00106 (18)	0.09283 (13)	0.21156 (12)	0.0303 (3)	
C2	0.09212 (18)	0.12971 (14)	0.28796 (12)	0.0303 (3)	
C3	−0.0038 (2)	0.16117 (19)	0.39080 (14)	0.0413 (3)	
H3	−0.1233	0.1536	0.4137	0.050*	
C4	0.0758 (2)	0.2035 (2)	0.45931 (14)	0.0440 (4)	
H4	0.0090	0.2227	0.5283	0.053*	
C5	0.2529 (2)	0.21823 (16)	0.42790 (13)	0.0354 (3)	
C6	0.3480 (2)	0.18731 (19)	0.32456 (14)	0.0420 (3)	
H6	0.4670	0.1966	0.3010	0.050*	
C7	0.2693 (2)	0.14291 (18)	0.25570 (14)	0.0388 (3)	
H7	0.3362	0.1219	0.1874	0.047*	
C8	0.3378 (3)	0.2695 (2)	0.50365 (16)	0.0508 (4)	
C9	0.14714 (17)	0.27411 (13)	−0.06047 (12)	0.0293 (3)	
H9	0.2224	0.2129	−0.0154	0.035*	
C10	0.17772 (17)	0.41344 (13)	−0.10698 (12)	0.0295 (3)	
C11	0.0657 (2)	0.50459 (15)	−0.17403 (14)	0.0401 (3)	
H11	0.0817	0.5989	−0.2066	0.048*	
C12	−0.0706 (2)	0.45268 (17)	−0.19165 (16)	0.0450 (4)	
H12	−0.1478	0.5117	−0.2363	0.054*	
C13	−0.09077 (19)	0.31202 (16)	−0.14208 (14)	0.0369 (3)	
H13	−0.1824	0.2780	−0.1548	0.044*	
C14	0.32848 (19)	0.45316 (15)	−0.07695 (13)	0.0340 (3)	
C15A	0.5284 (5)	0.2569 (7)	0.4716 (4)	0.0837 (17)	0.631 (5)
H15A	0.5709	0.2910	0.5229	0.126*	0.631 (5)
H15B	0.5752	0.1600	0.4785	0.126*	0.631 (5)
H15C	0.5648	0.3115	0.3924	0.126*	0.631 (5)
C16A	0.2846 (6)	0.1834 (5)	0.6339 (3)	0.0748 (12)	0.631 (5)
H16A	0.1591	0.1899	0.6574	0.112*	0.631 (5)
H16B	0.3333	0.0867	0.6420	0.112*	0.631 (5)
H16C	0.3288	0.2208	0.6825	0.112*	0.631 (5)
C17A	0.2534 (7)	0.4241 (4)	0.5014 (5)	0.0908 (16)	0.631 (5)
H17A	0.1279	0.4279	0.5229	0.136*	0.631 (5)
H17B	0.2935	0.4546	0.5558	0.136*	0.631 (5)
H17C	0.2869	0.4845	0.4240	0.136*	0.631 (5)
C15B	0.4753 (14)	0.3681 (10)	0.4200 (7)	0.091 (3)	0.369 (5)
H15D	0.5405	0.3276	0.3578	0.137*	0.369 (5)
H15E	0.4156	0.4587	0.3877	0.137*	0.369 (5)
H15F	0.5542	0.3784	0.4632	0.137*	0.369 (5)
C16B	0.4717 (11)	0.1305 (9)	0.5576 (7)	0.082 (2)	0.369 (5)
H16D	0.5515	0.0987	0.4946	0.122*	0.369 (5)
H16E	0.5368	0.1557	0.6023	0.122*	0.369 (5)
H16F	0.4044	0.0564	0.6073	0.122*	0.369 (5)
C17B	0.2288 (10)	0.3074 (12)	0.5999 (7)	0.090 (3)	0.369 (5)
H17D	0.2967	0.3374	0.6399	0.135*	0.369 (5)
H17E	0.1412	0.3830	0.5747	0.135*	0.369 (5)
H17F	0.1729	0.2281	0.6518	0.135*	0.369 (5)

### Atomic displacement parameters (Å^2^)

**Table:**

	*U*^11^	*U*^22^	*U*^33^	*U*^12^	*U*^13^	*U*^23^
Co1	0.02694 (14)	0.02236 (13)	0.03728 (15)	−0.00481 (9)	−0.01280 (10)	−0.01105 (10)
O1	0.0366 (5)	0.0437 (6)	0.0567 (6)	0.0021 (4)	−0.0211 (5)	−0.0259 (5)
O2	0.0361 (5)	0.0291 (5)	0.0401 (5)	−0.0013 (4)	−0.0162 (4)	−0.0154 (4)
O3	0.0533 (7)	0.0294 (5)	0.0910 (10)	−0.0043 (5)	−0.0420 (7)	−0.0158 (6)
O4	0.0302 (5)	0.0389 (5)	0.0487 (6)	−0.0064 (4)	−0.0110 (4)	−0.0197 (5)
O5	0.0414 (7)	0.0568 (8)	0.0854 (10)	−0.0030 (6)	−0.0214 (6)	−0.0372 (8)
N1	0.0303 (5)	0.0258 (5)	0.0378 (6)	−0.0059 (4)	−0.0104 (4)	−0.0106 (4)
N2	0.0476 (7)	0.0267 (6)	0.0647 (9)	−0.0104 (5)	−0.0248 (7)	−0.0099 (6)
C1	0.0350 (6)	0.0248 (6)	0.0360 (6)	−0.0051 (5)	−0.0140 (5)	−0.0089 (5)
C2	0.0351 (6)	0.0260 (6)	0.0353 (6)	−0.0028 (5)	−0.0144 (5)	−0.0111 (5)
C3	0.0325 (7)	0.0567 (10)	0.0414 (7)	−0.0098 (6)	−0.0072 (6)	−0.0207 (7)
C4	0.0404 (8)	0.0611 (10)	0.0385 (7)	−0.0075 (7)	−0.0064 (6)	−0.0260 (7)
C5	0.0401 (7)	0.0365 (7)	0.0369 (7)	−0.0065 (6)	−0.0135 (6)	−0.0147 (6)
C6	0.0333 (7)	0.0573 (10)	0.0456 (8)	−0.0113 (6)	−0.0085 (6)	−0.0248 (7)
C7	0.0359 (7)	0.0482 (8)	0.0405 (7)	−0.0050 (6)	−0.0081 (6)	−0.0241 (6)
C8	0.0584 (10)	0.0606 (11)	0.0498 (9)	−0.0158 (8)	−0.0206 (8)	−0.0256 (8)
C9	0.0306 (6)	0.0234 (6)	0.0379 (6)	−0.0043 (5)	−0.0129 (5)	−0.0087 (5)
C10	0.0327 (6)	0.0237 (6)	0.0358 (6)	−0.0055 (5)	−0.0092 (5)	−0.0109 (5)
C11	0.0482 (8)	0.0241 (6)	0.0502 (8)	−0.0063 (6)	−0.0196 (7)	−0.0046 (6)
C12	0.0478 (8)	0.0339 (7)	0.0568 (9)	−0.0013 (6)	−0.0302 (7)	−0.0036 (7)
C13	0.0346 (7)	0.0348 (7)	0.0476 (8)	−0.0063 (5)	−0.0178 (6)	−0.0112 (6)
C14	0.0348 (7)	0.0276 (6)	0.0445 (7)	−0.0075 (5)	−0.0111 (6)	−0.0126 (5)
C15A	0.0529 (18)	0.143 (5)	0.089 (3)	−0.029 (2)	−0.0157 (17)	−0.069 (3)
C16A	0.093 (3)	0.098 (3)	0.0494 (16)	−0.026 (2)	−0.0322 (17)	−0.0201 (17)
C17A	0.129 (4)	0.059 (2)	0.123 (4)	−0.003 (2)	−0.069 (3)	−0.049 (2)
C15B	0.136 (6)	0.098 (5)	0.075 (4)	−0.070 (5)	−0.032 (4)	−0.030 (3)
C16B	0.084 (4)	0.098 (5)	0.084 (4)	−0.007 (3)	−0.053 (4)	−0.029 (3)
C17B	0.077 (4)	0.144 (8)	0.087 (4)	−0.002 (4)	−0.026 (3)	−0.085 (6)

### Geometric parameters (Å, º)

**Table:**

Co1—O2	2.1252 (9)	C8—C15B	1.566 (8)
Co1—O2^i^	2.1252 (9)	C8—C16B	1.658 (8)
Co1—O4	2.1104 (11)	C8—C17B	1.382 (7)
Co1—O4^i^	2.1103 (11)	C9—H9	0.9300
Co1—N1	2.1638 (11)	C10—C9	1.3863 (18)
Co1—N1^i^	2.1638 (11)	C10—C11	1.384 (2)
O1—C1	1.2526 (17)	C10—C14	1.4994 (18)
O2—C1	1.2702 (16)	C14—N2	1.3224 (18)
O3—C14	1.2335 (19)	C11—C12	1.383 (2)
O4—H41	0.87 (3)	C11—H11	0.9300
O4—H42	0.84 (2)	C12—H12	0.9300
O5—H51	0.81 (3)	C13—C12	1.382 (2)
O5—H52	0.86 (3)	C13—H13	0.9300
N1—C9	1.3357 (16)	C15A—H15A	0.9600
N1—C13	1.3375 (18)	C15A—H15B	0.9600
N2—H21	0.83 (2)	C15A—H15C	0.9600
N2—H22	0.85 (2)	C16A—H16A	0.9600
C1—C2	1.5028 (17)	C16A—H16B	0.9600
C2—C3	1.389 (2)	C16A—H16C	0.9600
C2—C7	1.383 (2)	C17A—H17A	0.9600
C3—C4	1.379 (2)	C17A—H17B	0.9600
C3—H3	0.9300	C17A—H17C	0.9600
C4—H4	0.9300	C15B—H15D	0.9600
C5—C4	1.386 (2)	C15B—H15E	0.9600
C5—C6	1.390 (2)	C15B—H15F	0.9600
C5—C8	1.534 (2)	C16B—H16D	0.9600
C6—C7	1.389 (2)	C16B—H16E	0.9600
C6—H6	0.9300	C16B—H16F	0.9600
C7—H7	0.9300	C17B—H17D	0.9600
C8—C15A	1.455 (4)	C17B—H17E	0.9600
C8—C16A	1.560 (4)	C17B—H17F	0.9600
C8—C17A	1.564 (4)		
			
O2^i^—Co1—O2	180.0	C17B—C8—C15B	119.4 (6)
O2—Co1—N1	87.77 (4)	C17B—C8—C16B	105.4 (5)
O2^i^—Co1—N1	92.23 (4)	N1—C9—C10	123.81 (12)
O2—Co1—N1^i^	92.23 (4)	N1—C9—H9	118.1
O2^i^—Co1—N1^i^	87.77 (4)	C10—C9—H9	118.1
O4—Co1—O2	86.55 (4)	C9—C10—C14	116.42 (12)
O4^i^—Co1—O2	93.45 (4)	C11—C10—C9	118.10 (12)
O4—Co1—O2^i^	93.45 (4)	C11—C10—C14	125.46 (12)
O4^i^—Co1—O2^i^	86.55 (4)	C10—C11—H11	120.7
O4^i^—Co1—O4	180.0	C12—C11—C10	118.68 (13)
O4—Co1—N1	91.29 (4)	C12—C11—H11	120.7
O4^i^—Co1—N1	88.71 (4)	C11—C12—H12	120.4
O4—Co1—N1^i^	88.71 (4)	C13—C12—C11	119.21 (14)
O4^i^—Co1—N1^i^	91.29 (4)	C13—C12—H12	120.4
N1^i^—Co1—N1	180.0	N1—C13—C12	122.83 (13)
C1—O2—Co1	123.73 (8)	N1—C13—H13	118.6
Co1—O4—H41	99.1 (18)	C12—C13—H13	118.6
Co1—O4—H42	129.8 (16)	O3—C14—N2	122.61 (14)
H42—O4—H41	112 (2)	O3—C14—C10	119.44 (12)
H51—O5—H52	111 (3)	N2—C14—C10	117.94 (13)
C9—N1—Co1	117.05 (9)	C8—C15A—H15A	109.5
C9—N1—C13	117.36 (12)	C8—C15A—H15B	109.5
C13—N1—Co1	125.54 (9)	C8—C15A—H15C	109.5
C14—N2—H21	122.6 (14)	H15A—C15A—H15B	109.5
C14—N2—H22	115.3 (15)	H15A—C15A—H15C	109.5
H21—N2—H22	122 (2)	H15B—C15A—H15C	109.5
O1—C1—O2	124.68 (12)	C8—C16A—H16A	109.5
O1—C1—C2	116.71 (12)	C8—C16A—H16B	109.5
O2—C1—C2	118.57 (12)	C8—C16A—H16C	109.5
C3—C2—C1	119.37 (13)	H16A—C16A—H16B	109.5
C7—C2—C1	122.20 (13)	H16A—C16A—H16C	109.5
C7—C2—C3	118.28 (12)	H16B—C16A—H16C	109.5
C2—C3—H3	119.6	C8—C17A—H17A	109.5
C4—C3—C2	120.84 (14)	C8—C17A—H17B	109.5
C4—C3—H3	119.6	C8—C17A—H17C	109.5
C3—C4—C5	121.64 (14)	H17A—C17A—H17B	109.5
C3—C4—H4	119.2	H17A—C17A—H17C	109.5
C5—C4—H4	119.2	H17B—C17A—H17C	109.5
C4—C5—C6	117.23 (13)	C8—C15B—H15D	109.5
C4—C5—C8	120.78 (14)	C8—C15B—H15E	109.5
C6—C5—C8	121.97 (14)	C8—C15B—H15F	109.5
C5—C6—H6	119.2	H15D—C15B—H15E	109.5
C7—C6—C5	121.52 (14)	H15D—C15B—H15F	109.5
C7—C6—H6	119.2	H15E—C15B—H15F	109.5
C2—C7—C6	120.48 (13)	C8—C16B—H16D	109.5
C2—C7—H7	119.8	C8—C16B—H16E	109.5
C6—C7—H7	119.8	C8—C16B—H16F	109.5
C5—C8—C16A	109.22 (19)	H16D—C16B—H16E	109.5
C5—C8—C17A	107.8 (2)	H16D—C16B—H16F	109.5
C5—C8—C15B	108.0 (3)	H16E—C16B—H16F	109.5
C5—C8—C16B	104.1 (3)	C8—C17B—H17D	109.5
C15A—C8—C5	114.7 (2)	C8—C17B—H17E	109.5
C15A—C8—C16A	107.7 (3)	C8—C17B—H17F	109.5
C15A—C8—C17A	112.0 (3)	H17D—C17B—H17E	109.5
C16A—C8—C17A	104.9 (3)	H17D—C17B—H17F	109.5
C15B—C8—C16B	100.0 (6)	H17E—C17B—H17F	109.5
C17B—C8—C5	117.3 (3)		
			
Co1—O2—C1—O1	14.57 (19)	C4—C5—C8—C17A	−64.5 (3)
Co1—O2—C1—C2	−163.00 (9)	C4—C5—C8—C15B	−142.0 (5)
Co1—N1—C9—C10	178.01 (10)	C4—C5—C8—C16B	112.4 (4)
C13—N1—C9—C10	0.2 (2)	C4—C5—C8—C17B	−3.5 (6)
Co1—N1—C13—C12	−177.98 (13)	C6—C5—C8—C15A	−11.4 (4)
C9—N1—C13—C12	−0.4 (2)	C6—C5—C8—C16A	−132.3 (3)
O1—C1—C2—C3	27.3 (2)	C6—C5—C8—C17A	114.2 (3)
O1—C1—C2—C7	−148.24 (15)	C6—C5—C8—C15B	36.7 (5)
O2—C1—C2—C3	−154.98 (14)	C6—C5—C8—C16B	−68.9 (4)
O2—C1—C2—C7	29.5 (2)	C6—C5—C8—C17B	175.2 (6)
C1—C2—C3—C4	−176.12 (15)	C5—C6—C7—C2	0.7 (3)
C7—C2—C3—C4	−0.4 (2)	C11—C10—C9—N1	0.0 (2)
C1—C2—C7—C6	175.20 (15)	C14—C10—C9—N1	178.41 (13)
C3—C2—C7—C6	−0.4 (2)	C9—C10—C11—C12	−0.1 (2)
C2—C3—C4—C5	0.9 (3)	C14—C10—C11—C12	−178.35 (15)
C6—C5—C4—C3	−0.5 (3)	C9—C10—C14—O3	11.8 (2)
C8—C5—C4—C3	178.25 (17)	C9—C10—C14—N2	−167.08 (14)
C4—C5—C6—C7	−0.3 (3)	C11—C10—C14—O3	−169.96 (16)
C8—C5—C6—C7	−179.05 (17)	C11—C10—C14—N2	11.2 (2)
C4—C5—C8—C15A	169.9 (3)	C10—C11—C12—C13	0.0 (3)
C4—C5—C8—C16A	49.0 (3)	N1—C13—C12—C11	0.3 (3)

Symmetry code: (i) −*x*, −*y*, −*z*.

### Hydrogen-bond geometry (Å, º)

**Table:**

*D*—H···*A*	*D*—H	H···*A*	*D*···*A*	*D*—H···*A*
N2—H21···O1^ii^	0.82 (2)	2.15 (2)	2.935 (2)	159 (2)
N2—H22···O3^iii^	0.85 (2)	2.07 (2)	2.907 (2)	166 (2)
O4—H41···O1^i^	0.87 (3)	1.79 (3)	2.6230 (17)	160 (3)
O4—H42···O5	0.84 (2)	2.01 (2)	2.852 (2)	176.4 (19)
O5—H51···O3	0.82 (3)	2.14 (3)	2.942 (2)	164 (3)
O5—H52···O2^iv^	0.87 (3)	2.17 (3)	3.0331 (19)	175 (3)

Symmetry codes: (i) −*x*, −*y*, −*z*; (ii) −*x*, −*y*+1, −*z*; (iii) −*x*+1, −*y*+1, −*z*; (iv) −*x*+1, −*y*, −*z*.
